# Transcriptome analysis of avian reovirus-mediated changes in gene expression of normal chicken fibroblast DF-1 cells

**DOI:** 10.1186/s12864-017-4310-5

**Published:** 2017-11-25

**Authors:** Xiaosai Niu, Yuyang Wang, Min Li, Xiaorong Zhang, Yantao Wu

**Affiliations:** Jiangsu Co-Innovation Center for Prevention of Animal Infectious Diseases and Zoonoses, College of Veterinary Medicine, Yangzhou University, 48 East Wenhui Road, Yangzhou, Jiangsu 225009 China

**Keywords:** Avian reovirus, RNA-Seq, Transcriptome, Anti-viral responses

## Abstract

**Background:**

Avian reovirus (ARV) is an important poultry pathogen that can cause immunosuppression. In this study, RNA-Seq technology was applied to investigate the transcriptome-wide changes of DF-1 cells upon ARV infection at the middle stage.

**Results:**

Total RNA of ARV-infected or mock-infected samples at 10 and 18 h post infection (hpi) was extracted to build RNA-Seq datasets. Analysis of the sequencing data revealed that the expressions of numerous genes were altered, and a panel of differentially expressed genes were confirmed with RT-qPCR. At 10 hpi, 104 genes were down-regulated and 64 were up-regulated, while the expressions of 47 genes were increased and only one was down-regulated, which may play a role in retinoic acid biosynthesis, at 18 hpi in the ARV-infected cells. The similar profiles of up-regulated genes between the two groups of infected cells suggest that ARV infection activated a prolonged antiviral response of host cells. Alternative splicing analysis found no significantly changed events altered by ARV infection.

**Conclusions:**

Overall, the differential expression profile presented in this study can be used to expand our understanding of the comprehensive interactions between ARV and the host cells, and may be helpful for us to reveal the pathogenic mechanism on the molecular level.

**Electronic supplementary material:**

The online version of this article (10.1186/s12864-017-4310-5) contains supplementary material, which is available to authorized users.

## Background

Avian reovirus (ARV) is member of the *Orthoreovirus* genus that has recently been classified into the *Spinareovirinae* subfamily, which is one of two subfamilies in the *Reoviridae* family [1]. ARV is an important pathogen of birds and has been impacting poultry for nearly 60 years since it was first detected in 1957 [2, 3], and it is still prevalent in poultry until now, causing considerable economical loss in the global poultry industry [4–6]. Efficient and simple detecting methods may be helpful to control and prevent ARV infection [7]. Horizontal transmission is the main route of infection, with infrequent egg transmission [8]. Though ARV was found to be ubiquitous in poultry flocks, several strains could cause severe diseases [9]. These pathogenic strains can cause tenosynovitis individually [9], and additionally usually cause mixed infections together with other pathogens, such as chicken anemia agent [10, 11]. It has been demonstrated that ARV can replicate in macrophages and cause immunosuppression [8, 12].

The pathogenicity and epizootiology of ARV have been well studied, but the pathogenesis at the molecular level is poorly understood. An excellent review on the structural and biological characteristics of ARV was published 10 years ago [8]. Though many researchers have done brilliant work to reveal the pathogenesis of ARV infection at the molecular level in recent years, several major questions raised in the review remain unresolved. Previous studies showed that σC and P10 can induce apoptosis in different ways [13, 14], and a subsequent study correlated ARV-induced apoptosis with tissue injury [15]. Another study demonstrated that ARV can induce autophagy to promote viral titer [16]. Subsequent studies revealed the connection between ARV-induced autophagy and apoptosis [17, 18]. It was also demonstrated that ARV disrupts many cellular pathways, regulating protein translation, cell proliferation, and cell metabolism [19–22]. However, these results are scattered and hard to reconcile. Some studies applied proteomic analysis and microarray analysis to get a comparatively integrated data set [23, 24]. However, these methods have several disadvantages compared with RNA-Seq. RNA-Seq now provides a way to investigate virus-mediated changes on the transcriptome of host cells, with high accuracy and low background [25, 26]. Additionally, it provides information on alternative splicing events, analyzing single nucleotide polymorphism, and predicting novel transcripts [27–29]. In this study, we tried to build a complete expression profile of ARV-mediated changes at the transcriptional level using RNA-Seq to unveil the complex interactions between ARV and host cells.

## Methods

### Cell culture and virus inoculation

Chicken embryonic fibroblast cell line DF-1 (CRL-12203, ATCC) cells were cultured with high glucose (4.5 g D-Glucose/L) Dulbecco’s Modified Eagle Medium (HG-DMEM) (Basal Media, Shanghai, China) supplemented with 10% (*v*/v) fetal bovine serum (FBS) (Gibco, Shanghai, China) at 37 °C and 5% CO_2_. The ARV strain GX/2010/1, causing severe tenosynovitis and enteritis, was isolated by our lab and propagated in chicken embryo fibroblasts (CEF) cells, and was reported to trigger autophagy in host cells to promote virus production [16]. The sequences of this strain are available in the GenBank database under the accession numbers KJ476699−KJ476708. The sixth generation of the purified virus was used in this study and the median tissue culture infective dose (TCID_50_) per milliliter (ml) of the virus was determined by the Reed-Muench method in CEF cells [30].

**Fig. 1:**
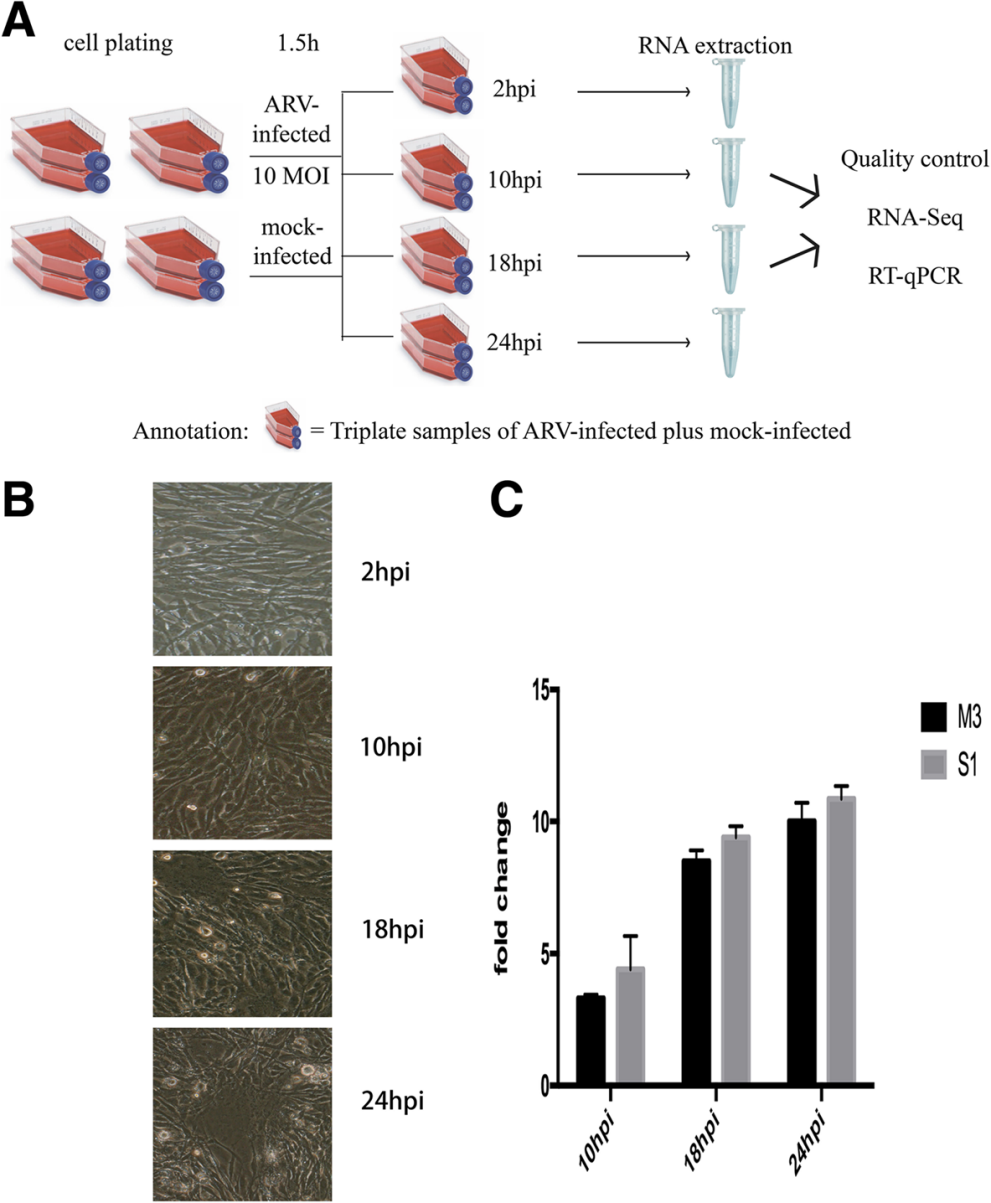
Overview of RNA-Seq approach. **a** Experimental setup for RNA-Seq datasets. **b** DF-1 cells were infected with ARV and the cytopathic effect was assessed at different time points. **c** The replication of ARV was monitored by RT-qPCR analysis of ARV genes M3 and S1

One day before virus inoculation, approximately 2 × 10^6^ DF-1 cells were seeded into 75 cm^2^ flasks (Corning, ME, USA). When monolayer was complete (approximately 7 × 10^6^ cells), culture medium was discarded and the cells were rinsed with phosphate buffered saline once. The purified virus was diluted to 10 multiplicity of infection (MOI) per 5 mL with HG-DMEM and applied into each flask of the ARV-infected group and an equal volume of HG-DMEM was added in the mock-infected group. After incubated at 37 °C for 1.5 h, the medium was changed to HG-DMEM supplemented with 2% FBS. Then the cells were continued to be incubated at 37 °C for 2, 10, 18 and 24 h (Fig. 1a).

### Total RNA extraction and cDNA library construction

At specified hours post infection (hpi), the medium was discarded and total RNA was extracted from triplicate samples of uninfected or ARV infected groups using the Ultrapure RNA Kit (CWBIO, Beijing, China) according to manufacturer’s protocol. RNA degradation and contamination was monitored on 1% agarose gels. RNA purity was checked using the NanoPhotometer® spectrophotometer (IMPLEN, CA, USA). RNA concentration was measured using Qubit® RNA Assay Kit in Qubit® 2.0 Flurometer (Life Technologies, CA, USA). RNA integrity was assessed using the RNA Nano 6000 Assay Kit of the Bioanalyzer 2100 system (Agilent Technologies, CA, USA).

A total amount of 3 μg RNA per sample was used as input material for the RNA sample preparations. Sequencing libraries were generated using NEBNext® Ultra™ RNA Library Prep Kit for Illumina® (NEB, USA) following the manufacturer’s recommendations, and index codes were added to attribute sequences to each sample.

### Clustering and sequencing

The clustering of the index-coded samples was performed on a cBot Cluster Generation System using TruSeq PE Cluster Kit v3-cBot-HS (Illumia) according to the manufacturer’s instructions. After cluster generation, the library preparations were sequenced on an Illumina Hiseq platform and 150 bp paired-end reads were generated. Raw reads of fastq format were first processed through custom written Perl scripts. At the same time, Q20, Q30, and GC content were calculated. All of the downstream analyses were based on clean, high quality data.

### Reads mapping and quantification of gene expression level

The index of the chicken reference genome (Ensembl, Galgal4, updated 11-2015) was built using Bowtie v2.2.3 [31] and paired-end clean reads were aligned to the reference genome using TopHat v2.0.12 [32]. And HTSeq v0.6.1 was used to count the number of reads mapped to each gene [33]. Then, the expression level of each gene was calculated by the expected Fragments Per Kilobase of transcript per Million fragments mapped (FPKM) [34].

### Differential expression analysis

Differential expression analysis of two groups was performed using the DESeq R package (1.18.0) [35]. DESeq provides statistical routines for determining differential expression in digital gene expression data using a model based on a negative binomial distribution. The resulting *P*-values were adjusted using the Benjamini and Hochberg’s approach for controlling the false discovery rate [36]. Genes with an adjusted *P*-value < 0.05 found by DESeq were considered to be differentially expressed. Additionally, KOBAS 2.0 software was used to test the statistical enrichment of differentially expressed genes (DEGs) in the Kyoto Encyclopedia of Genes and Genomes (KEGG) pathways [37].

### RT-qPCR

Reverse transcription-quantitative polymerase chain reaction (RT-qPCR) was carried out based on the basic rules of the MIQE guidelines [38]. Briefly, 5 μg of total RNA (described above) was reverse transcribed using M-MLV reverse transcriptase (Transgen, Beijing, China) with a random hexamer primer (Genscript, Nanjing, China). The mixtures were diluted 1:10 with nuclease free water and then used as templates for qPCR. The qPCR analysis was performed using AceQ® qPCR SYBR® Green Master Mix (Vazyme, Nanjing, China) with 250 nM forward and reverse primers (Additional file 1). The reaction was carried out using LightCycler® Nano (Roche) with the following cycling conditions: an initial denaturation at 95 °C for 600 s followed by 45 cycles of 95 °C for 10 s and 60 °C for 30 s. Fold change was determined by the 2^-△△Ct^ method [39].

### Novel transcripts and alternative splicing prediction

The Cufflinks v2.1.1 Reference Annotation Based Transcript (RABT) assembly method was used to construct and identify both known and novel transcripts from TopHat alignment results [40]. Alternative splicing (AS) events were classified to five major types by the software rMATS (Multivariate Analysis of Transcript Splicing) v3.2.5 [41]. The number of AS events in each sample was estimated separately. Because the chicken genome has been recently updated [42], the differentially expressed novel transcripts were retrieved by the BLAST tool on National Center for Biotechnology Information (NCBI).

## Results

### ARV infection of DF-1 cells and viral replication dynamics

To further study the molecular mechanism of ARV infection, DF-1 cells were infected with the virus for different time points at 10 MOI. The high dosage of virus was used to overcome the influence of uninfected cells [43, 44]. Infection and mock-infection were performed in biological triplicate for each time point and total RNA was extracted from both groups. The replication of the viral genome was determined by RT-qPCR and the fold change of M3 and S1 showed similar trends (Fig. 1c). Cytopathic effects could be seen at 18 hpi (Fig. 1b). To obtain an obviously changed transcriptome profile and minimize the influence of cell death and lysis, data at 2 hpi and 24 hpi were discarded and the remaining two groups were analyzed by RNA-Seq. One uninfected 10 hpi sample was lost, leaving a final total of 11 samples that were sequenced.

### RNA-Seq results

After an overall quality review, mRNA was purified from total RNA using poly-T oligo-attached magnetic beads, and then the cDNA library was constructed, with quality assessment. After cluster generation, the library preparations were sequenced on an Illumina Hiseq platform and 150 bp paired-end reads were generated. The sequence run of each sample yielded at least 42 million clean reads and the lowest value of the reads possessing a Q-score > 20 was 95%, and the bottom line of the reads with a Q-score > 30 was 88% (Table 1). These results meet the requirements that more than 10 million reads are needed to construct a high quality eukaryotic transcriptome for discovering new genes and transcripts [45]. Importantly, all samples had between 71.81% and 77.45% of total reads mapped to the chicken reference genome, and the percentage of uniquely mapped reads was between 70.75% and 76.26% (Table 1).

**Table 1:** RNA-Seq overview

Sample name	Raw reads	Clean reads	Q20 (%)	Q30 (%)	Total mapped (%)	Uniquely mapped (%)
V10_1	53,944,714	52,820,194	97.37	93.68	75.82	74.70
V10_2	59,160,706	57,378,292	97.39	93.81	76.44	75.46
V10_3	53,875,186	51,827,434	97.17	93.2	77.45	76.26
NC10_1	57,025,992	54,713,970	96.84	92.51	77.09	75.89
NC10_2	60,174,142	58,164,444	95.01	88.06	71.81	70.75
V18_1	58,591,660	56,688,518	95.1	88.24	72.40	71.34
V18_2	63,030,008	60,813,608	95.05	88.16	71.95	70.89
V18_3	44,002,734	42,770,420	96.75	92.22	73.96	72.89
NC18_1	51,089,720	48,895,588	96.24	91.26	74.02	72.96
NC18_2	61,366,046	59,972,578	97.11	93.2	75.05	73.90
NC18_3	68,023,822	65,597,914	97.36	93.79	76.15	75

*Abbreviations: V* ARV-infected, *NC* mock-infected

**Fig. 2:**
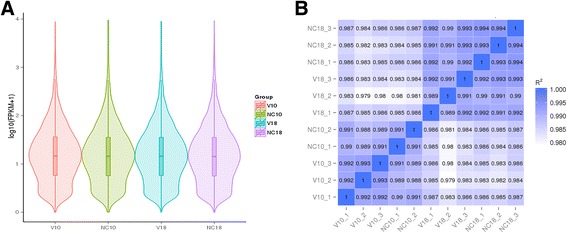
The quality assessment of RNA-Seq. **a** Violin diagram of the distribution of average FPKM values per sample group. **b** The correlation between all the samples shown as a squared Pearson correlation coefficient (R^2^)

Then, the mapped data were used to predict the novel transcripts and analyze the five major types of AS events, including SE (skipped exon), A5SS (alternative 5′ splice site), A3SS (alternative 3′ splice site), MXE (mutually exclusive exon), and RI (retained intron). The predicted novel genes were further analyzed together with the known genes. No significantly changed AS events were found between each group. The mapped data was normalized by calculating the FPKM and the distribution of mean FPKM per gene was found to be uniform between the four conditions (Fig. 2a). The correlation of gene expression levels between all of the samples was investigated using the squared Pearson correlation coefficient (R^2^), and the minimum value was 0.979 (Fig. 2b). These results indicate that the expression levels of different genes or groups of genes are comparable, suggesting that the treatment is repeatable and has little variation. Therefore, the accuracy of the subsequent analysis of differentially expressed genes is likely to be high. Because the transcripts of the ARV genome do not have a poly-A 3′ tail [8], there are no reads can be used to indicate the replication of ARV.

### Differentially expressed genes upon ARV infection

**Fig. 3:**
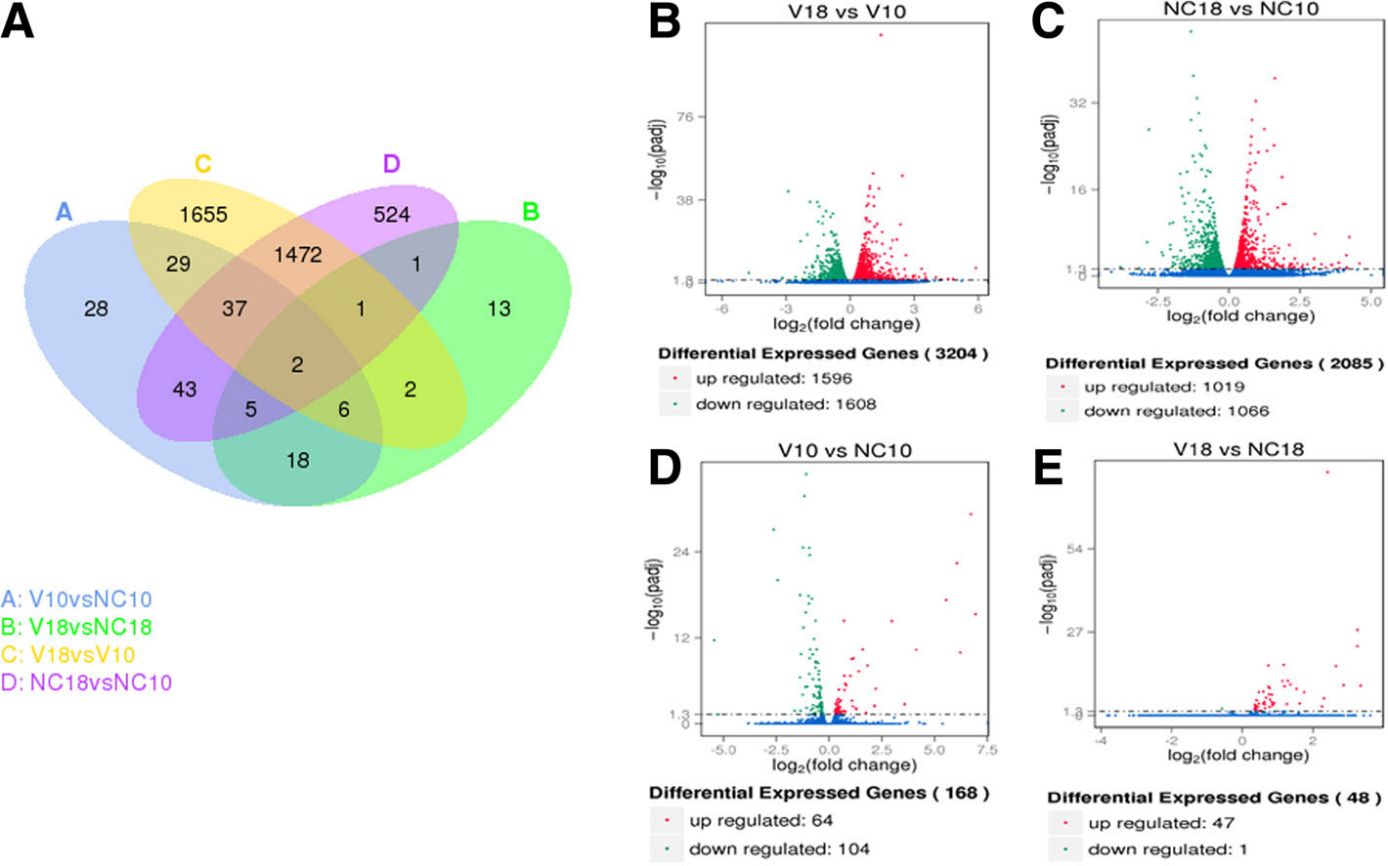
Overall analysis of ARV-mediated changes in gene expression. **a** Venn diagram of DEGs between all samples. **b**−**e** Volcano plots of DEGs from ARV-infected cells at 10 hpi to 18 hpi (**b**), mock-infected cells at 10 hpi to 18 hpi (**c**), and ARV-infected cells to mock-infected cells at each time point (**d**, **e**)

To further investigate the differential expression patterns in DF-1 cells between infected and mock-infected samples, the normalized gene expression level data were analyzed by DESeq. The resulting *P*-values were adjusted after correction for multiple testing and the DEGs were defined by having adjusted P-values (padj) < 0.05. The infected and mock-infected were compared with each other and the outline of the DEGs can be seen in Fig. 3a. Though there were significant alterations in the pairwise comparisons of different time points in the infected samples (Fig. 3b), the corresponding mock-infected samples also had big differences (Fig. 3c). This interference might result from cell culture and so should be discarded in the future studies. All of the DEGs were clustered and the results exhibited a clear time-dependent change in gene expression (Additional file 2).

The distinct effect on gene expression upon ARV infection was carefully examined. Compared with mock infected controls, 168 changes in the transcriptome, with 64 up-regulated and 104 down-regulated DEGs, were observed in response to ARV infection at 10 hpi (Fig. 3d, Additional file 3). Interestingly, only 47 up-regulated DEGs, with 31 genes in accordance with the 10 hpi group, and a single down-regulated novel gene were identified at 18 hpi (Fig. 3e, Additional file 3). These novel genes among the DEGs were retrieved by the BLAST tool. In addition, the pathway enrichment result can be seen at additional file 4. The DEGs with a fold change larger than 2 are listed in Table 2 with UniProtKB Keywords annotation [46], as a 2-fold threshold is commonly used to indicate biological significance [47].

**Table 2:** DEGs and the respective biological process in DF-1 cells upon ARV infection. DEGs with fold change > 2 are listed

	Gene-id	Gene-name	Description	Keywords of biological process	Fold change	
					10 hpi	18 hpi
Up	ENSGALG00000016400	RSAD2	Radical S-adenosyl methionine domain containing 2	antiviral defense, innate immunity, et al.	6.9368	3.2553
	ENSGALG00000006384	IFIT5	Interferon induced protein with tetratricopeptide repeats 5	antiviral defense, innate immunity, et al.	6.7163	3.3529
	ENSGALG00000013723	OASL	2′-5′-oligoadenylate synthetase-like	antiviral defense, innate immunity, et al.	6.2188	3.2579
	ENSGALG00000013575	ISG12(2)	Interferon-stimulated genes 12 (2)	–	6.0596	2.2561
	ENSGALG00000016142	Mx	Myxovirus resistance	antiviral defense, innate immunity, et al.	5.5442	2.653
	ENSGALG00000028982	CMPK2	Cytidine monophosphate (UMP-CMP) kinase 2	Pyrimidine biosynthesis	4.1368	2.3091
	ENSGALG00000009479	SAMD9L	Sterile alpha motif domain containing protein 9-like	endosomal vesicle fusion	3.5798	2.8676
	ENSGALG00000006138	HELZ2	Helicase with zinc finger domain 2	Transcription regulation	2.9797	2.4167
	ENSGALG00000016964	EPSTI1	Epithelial stromal interaction 1	–	2.2115	1.7528
	Novel00328	predicted: TRIM25-like	Tripartite motif containing 25	antiviral defense, innate immunity, et al.	2.1606	–
	Novel00773	predicted: EEA1-like	Early endosome antigen 1-like	endocytosis, vesicle fusion, et al.	2.1431	–
Down	ENSGALG00000010791	LEXM	Lymphocyte expansion molecule	positive regulation of cell proliferation, et al.	−5.4359	–
	ENSGALG00000000607	GPR37L1	G protein-coupled receptor 37 like 1	positive regulation of MAPK cascade, et al.	−5.2343	–
	ENSGALG00000002742	TMEM132B	Transmembrane protein 132B	–	−2.6179	–
	Novel01111	lncRNA	Uncharacterized LOC107053801	–	−2.4302	–

**Fig. 4:**
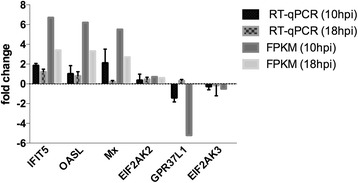
Confirmation of DEGs by RT-qPCR. Expression of four up-regulated genes and two down-regulated genes was confirmed by RT-qPCR. The results are presented as fold change of ARV-infected cells compared to mock-infected cells at the indicate time point. Fold change using the FPKM was included for comparison of expression pattern

Four up-regulated DEGs and two down-regulated DEGs were selected to be validated with RT-qPCR. The results show a similar pattern of ARV-mediated changes as was seen in the DEG analysis of RNA-Seq data (Fig. 4).

## Discussion

ARV is one of the major pathogens that can cause immunosuppression in poultry [7]. Though the pathology and some molecular characteristics of ARV have been well studied [2, 8, 48], there are only a few reports that can be used to help us understand the molecular basis of ARV infection. In this study, the DEG listed in Table 2 show that DF-1 cells exerted a prolonged antiviral response upon ARV infection. The up-regulation of RSAD2 (radical S-adenosyl methionine domain containing 2), IFIT5 (interferon induced protein with tetratricopeptide repeats 5), OASL (2′-5′-oligoadenylate synthetase-like), ISG12(2) (interferon-stimulated genes) and Mx (myxovirus resistance) have been reported in the infection of infectious bursal disease virus (IBDV), which is another important pathogen similar to ARV but can cause much higher mortality and much more serious immunosuppression [43]. EPSTI1 (epithelial-stromal interaction 1) does not have a Gene Ontology (GO) annotation. A recent report indicated that EPSTI1 plays a key role in IL-28A (interferon-λ2) mediated antiviral activity [49]. These interferon- (IFN-) induced genes (ISGs) with high expression levels reflect the stimulation of IFNs. Even though no significant elevation of expression levels of IFN genes were identified in this study, the up-regulation of TLR3 (Toll-like receptor 3), MYD88 (Myeloid differentiation factor 88), IRF1 (IFN regulatory factor 1), and IRF3 (IFN regulatory factor 3) were found. TLR3 plays key roles in detecting virus-derived dsRNA and the TLR3 genes are polymorphic among different chicken breeds [50, 51]. In addition to TLR-induced pathways, members of the RLR family (retinoic acid inducible gene-I like receptor) constitute another TLR-independent anti-virus system. In our results, DHX58 (DEXH-box helicase 58, also known as LGP2, laboratory of genetics and physiology 2, or RLR3, RIG-I like receptor 3), IFIH1 (IFN-induced helicase C domain-containing protein 1, also known as MDA5, melanoma differentiation-associated protein 5), TRIM25 (Tripartite motif-containing protein 25), and a predicted TRIM25-like gene were found to be up-regulated at 10 hpi or at both 10 and 18 hpi time points. Chickens lack RIG-I (retinoic acid-inducible gene I), but the function of sensing viral infections can be performed by LGP2 and MDA5, which can interact with MAVS (mitochondrial antiviral signaling protein) or STING (stimulator of IFN genes) to stimulate the expression of IFNs [52–54].

DF-1 cells construct an antiviral environment through the expression of ISGs, including EIF2AK2 (Eukaryotic translation initiation factor 2-alpha kinase 2, also known as PKR, protein kinase RNA-activated). PKR is IFN-induced dsRNA-dependent enzymes. Active PKR can catalyze Ser-51 phosphorylation of the alpha subunit of EIF2, resulting in inhibition of protein synthesis at the initiation step of translation [55]. However, a previous report demonstrated that σA, an ARV encoded dsRNA binding protein, can block the activation of PKR and restore translation. In that report, the inhibition of vaccinia virus replication might reflect mechanisms other than OAS and PKR to be responsible for the antiviral effects [56]. Interestingly, another eukaryotic translation initiation factor 2-alpha kinase, EIF2AK3, was found to be down-regulated in our results. EIF2AK3, also known as PERK (PKR-like endoplasmic reticulum-resident kinase), is one of eIF2α kinases regulating gene expression in the unfolded protein response (UPR) and in amino acid starved cells [57]. Protein synthesis can be inhibited during viral infection due to ER stress triggered by UPR, and different viruses may adapt different strategies to interfere with the activity of PERK [58, 59]. The depressed expression of PERK may reflect that ARV can impair the stress response and activate protein translation in DF-1 cells. The regulation of the host cell translation system ensures efficient replication of ARV. There is also a hypothesis that gene expression of ARV is mainly regulated at the translational level, rather than transcriptional level [8]. The replication level of viral genome determined by RT-qPCR in our results is consistent with this hypothesis.

ARV was initially detected from the clinical case of tenosynovitis, and a direct link between the virus and disease had been conclusively demonstrated [2, 8]. Though this virus has been studied for many years, the molecular pathogenesis of the disease remains unclear. In our results, the elevated expression of a gene, WNT9a (also known as Wnt14), was observed, which might play a key role in the development of the disease. A previous report identified that Wnt14 plays a pivotal role in initiating synovial joint formation in the chick limb, but the researchers were unable to determine the specific pathway that is responsible for transducing the Wnt14 signal in joint formation [60]. Later, studies demonstrated that the Wnt/β-catenin signaling pathway is necessary and sufficient to induce early steps of synovial joint formation [61]. Subsequently, a precise expression pattern of various Wnts was analyzed during chick wing development [62]. Continued expression of Wnt14 in the mature joint might be good for the maintenance of joint integrity and was presumed to play a role in the etiology of rheumatoid arthritis in humans [60]. ARV can replicate and perhaps be persistent at hock joint of chicken [48]. The up-regulation of Wnt14, combined with the induction of apoptosis [15], may be responsible for ARV-induced joint damage and more severe tendon rupture.

## Conclusions

**Fig. 5:**
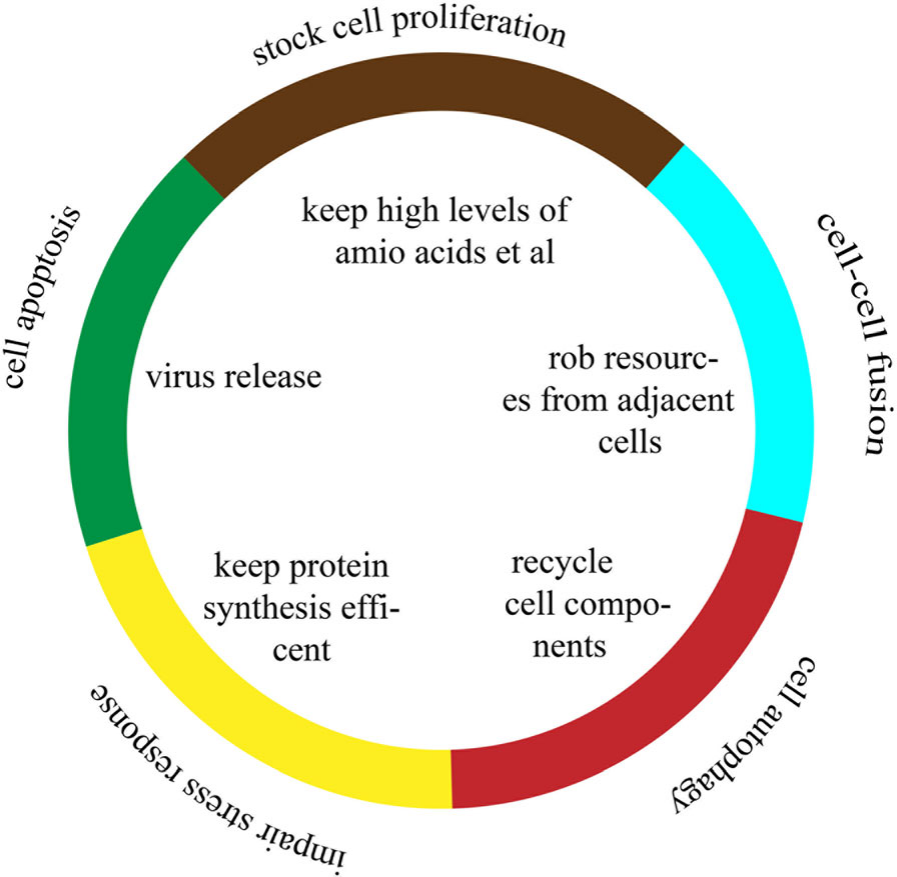
Diagram of the interactions between ARV and host cells

In conclusion, our results show that ARV infection stimulates a prolonged antiviral response in host cells and interferes with cell growth and cell death pathways. Our results also provide information that may be helpful to further investigate the pathogenesis of ARV infection. Combined with previous studies, we can begin to piece together the interactions between ARV and host cells (Fig. 5). However, the details of these interactions need to be further investigated in future studies.

## Additional files

Additional file 1: RT-qPCR Primers. All RT-qPCR primers for detection of the replication of ARV and the DEGs. (DOCX 46 kb)

Additional file 2: Cluster analysis of differentially expressed genes. Heatmap of the DEGs across all datasets based on log_10_ (FPKM + 1). (PDF 283 kb)

Additional file 3: Differentially expressed genes between sample groups. Sheet 1. The DEGs between mock- or ARV-infected samples at 10 hpi. Sheet 2. The DEGs between mock- or ARV-infected samples at 18 hpi. (XLSX 74 kb)

Additional file 4: KEGG pathway enrichment result. Sheet 1. The KEGG enrichment result of the DEGs between mock- or ARV-infected samples at 10hpi. Sheet 2. The KEGG enrichment result of the DEGs between mock- or ARV-infected samples at 18hpi. (XLSX 60 kb)

## Abbreviations

ARV: Avian reovirus
AS: Alternative splicing
Bp: Base pair
CEF: Chicken embryo fibroblasts
DEGs: Differentially expressed genes
FBS: Fetal bovine serum
FPKM: Fragments Per Kilobase of transcript per Million fragments mapped
GO: Gene Ontology
HG-DMEM: High glucose Dulbecco’s Modified Eagle Medium
Hpi: Hours post infection
IFN: Interferon
KEGG: Kyoto Encyclopedia of Genes and Genomes
MOI: Multiplicity of infection
Padj: Adjusted *P*-values
RNA-Seq: RNA sequencing
RT-qPCR: Reverse transcription-quantitative polymerase chain reaction
TCID_50_: Median tissue culture infective dose
