# Supplementary material for: Transcriptome analysis of avian reovirus-mediated changes in gene expression of normal chicken fibroblast DF-1 cells
Source: BMC Genomics. 2017 Nov 25;18:911. doi: 10.1186/s12864-017-4310-5 (PMC5702118; doi:10.1186/s12864-017-4310-5)
Supplement: Supplementary file 1 — RT-qPCR Primers. All RT-qPCR primers for detection of the replication of ARV and the DEGs. (DOCX 46 kb) [file 12864_2017_4310_MOESM1_ESM.docx]

**Table S1. Primers for RT-qPCR**

| Target of gene | Primers | Gene product |
| --- | --- | --- |
| IFIT5 | F: tggacaaggcacaagaggtt  R: ctcggtagcagagtcctagc | 90bp (794-883) |
| OASL | F: ggtctacgtgaagctgttgg  R: gtctttcagcttagcagggc | 109bp (519-627) |
| Mx | F: aaacgacctgatgttgcctg  R: aggaagagcaacaccagaca | 94bp (327-420) |
| EIF2AK2 | F: gctccctccagttgactaca  R: gaatacattggagcgtgggg | 114bp (282-395) |
| GPR37L1 | F: tacatgaagagcgcctggaa  R: ctcgttgaagatgaccacgg | 96bp (874-969) |
| EIF2AK3 | F: tacaaactgggcgaggatgt  R: acagccgagcagatgtactt | 101bp (496-596) |
| M3-μNS | F: cgtgtggaagcgttaaacca  R: tcatcacgctcgttcaggta | 95bp (1639-1733) |
| S1-σC | F: cgtatcattcacccgcgatt  R: tgttcgctgtaccatcacct | 113bp (831-943) |
