# Supplementary figures and images for: Transcriptome analysis of avian reovirus-mediated changes in gene expression of normal chicken fibroblast DF-1 cells
Source: BMC Genomics. 2017 Nov 25;18:911. doi: 10.1186/s12864-017-4310-5 (PMC5702118; doi:10.1186/s12864-017-4310-5)

Cluster analysis of differentially expressed genes

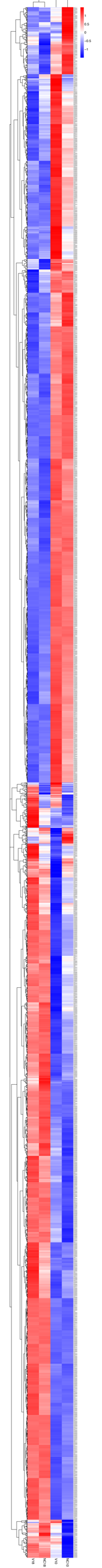

Supplement: Supplementary file 2 — Cluster analysis of differentially expressed genes. Heatmap of the DEGs across all datasets based on log10 (FPKM + 1). (PDF 283 kb) [file 12864_2017_4310_MOESM2_ESM.pdf]
